# Redox Status, Estrogen and Progesterone Production by Swine Granulosa Cells Are Impaired by Triclosan

**DOI:** 10.3390/ani12243559

**Published:** 2022-12-15

**Authors:** Giuseppina Basini, Francesca Grasselli, Fausto Quintavalla, Simona Bussolati, Valentina Andreoli, Alicia Maria Carrillo Heredero, Simone Bertini

**Affiliations:** Dipartimento di Scienze Medico-Veterinarie, Università degli Studi di Parma, 43126 Parma, Italy

**Keywords:** ovary, steroidogenesis, cell proliferation, free radicals, oxidative stress

## Abstract

**Simple Summary:**

Triclosan is widely used in cosmetics and hygiene products, including anti-bacterial hand sanitizers and disinfectants whose utilization has been greatly increased during the COVID-19 pandemic. Since critical health effects have been demonstrated for this substance we tested its potential harmful action on ovarian cells collected from pigs, a valuable animal model. We demonstrated that Triclosan impairs cell function, thus suggesting an impairment of reproductive function.

**Abstract:**

Triclosan is a chlorinated biphenolic with a broad spectrum of antiseptic activities used in cosmetics and hygiene products. Continuous exposure can lead to absorption and bioaccumulation of this substance with harmful health effects. In fact, previous studies have shown that Triclosan acts as an endocrine-disrupting chemical on reproductive organs, with consequent negative effects on reproductive physiology. Therefore, to assess potential adverse impacts on fertility, we tested Triclosan on swine granulosa cells, a model of endocrine reproductive cells. We examined its effects on the main features of granulosa cell functions such as cell growth (BrdU incorporation and ATP production) and steroidogenesis (17-β estradiol and progesterone secretion). Moreover, since oxidant–antioxidant balance plays a pivotal role in follicular function, redox status markers (superoxide, hydrogen peroxide and nitric oxide production, enzymatic and non-enzymatic scavenging activity) were studied. Our results show that Triclosan significantly inhibits cell growth (*p* < 0.001), steroidogenesis (*p* < 0.001), superoxide and nitric oxide production (*p* < 0.001), while it increases (*p* < 0.05) enzymatic defense systems. Collectively, these data suggest a disruption of the main granulosa cell functions, i.e., proliferation and hormone production, as well as an imbalance in redox status. On these bases, we can speculate that Triclosan would impair granulosa cell functions, thus exerting negative effects on reproductive function. Further studies are needed to explore lower Triclosan concentrations and to unravel its mechanisms of action at gene level.

## 1. Introduction

Triclosan, [5-chloro-2-(2,4-dichlorophenoxy) phenol] is a widely used disinfectant, preservative and antiseptic, whose harmful effects on health are not completely known. It is a biocide in use for more than 40 years to reduce or prevent the bacterial contamination of several products, primarily cosmetics and detergents, but also drugs, clothing, kitchen utensils, toys and furniture. Environmental contamination has been assessed worldwide. In particular, it has been documented that the Triclosan concentration in the United States’ surface water to is 2.3 μg/L [1], and 800 μg/kg in the sediment of Jamaica Bay. Furthermore, 86.161 μg/L was found in wastewater from a municipal sewage treatment plant in Savannah, United States [2,3], and 533 ng/L in Shanghai, China [4]. Moreover, in different Asian countries, such as Japan, South Korea and India, the concentration of Triclosan in water ranged from 0 to 5160 ng/L [5]. Unfortunately, environmental exposure to Triclosan not only disrupts microbiome but also increases antibiotic resistance [6]. As a matter of fact, Triclosan has been found in human tissues, including the liver and brain, as well as in the urine, breast milk and blood [7,8,9,10].

Exposure to Triclosan may occur mainly through oral intake and dermal contact. After oral ingestion, a rapid gastrointestinal absorption and median urinary excretion of 54% can occur within four days. Triclosan levels in plasma quickly increase, reaching the maximum concentration within 1–3 h. The terminal plasma half-life is 21 h and the major fraction is excreted within the first 24 h. [11]. The second main exposure route to Triclosan is dermal contact, with an absorption lower than 10% [12]. Triclosan is primarily excreted in urine as a glucuronide or sulfate conjugate [11]. Its half-life was reported to range from 1.3 to 1.4 d in water to 53.7 to 60.3 d in sediments [13]. The Triclosan bioaccumulation in organisms resulting from its strong lipophilicity has raised concerns; impairments of the thyroid, heart functions and neurodevelopment have been documented as well as the occurrence of metabolic disorders and an increased cancer risk [14]. Thus, in 2009 the American Public Health Association (APHA) recommended avoiding the use of Triclosan in personal care products [15]. The European Chemical Agency (ECHA) commented: “No safe use could be demonstrated for the proposed use of Triclosan” [16]. Moreover, in 2016 the U.S. Food and Drug Administration (FDA) banned the use of Triclosan in antibacterial soaps and body washes. This rule came into effect in September 2017 [17]. However, in the countries where Triclosan is widely used as a biocide, microbial resistance represents a great concern. In fact, it has recently been demonstrated that there is a decreased susceptibility and an acquired resistance to several disinfectants, including Triclosan [18]. Moreover, an emerging concern has been raised during the COVID-19 pandemic about the consequent use of non-alcohol-based anti-bacterial hand sanitizers and disinfectants [19] containing Triclosan as an antimicrobial or disinfecting agent. Since its virucidal efficacy had already been documented during the outbreak of severe acute respiratory syndrome (SARS) in 2003, Triclosan was also widely employed as a virucide against the SARS-CoV-2 strain [20]. Triclosan has been classified as an endocrine-disrupting chemical, and critical issues have been raised about its possible effects on reproductive functions [21,22,23]. In this context, a potential impairment of women’s reproductive health was recently reviewed by Haggerty et al. [23]. Recent studies on aquatic animals and rodents confirm the potential for Triclosan to act as an endocrine-disrupting chemical (EDC), mainly affecting the reproductive system. At present, however, the data about a disruption of the human endocrine system resulting from daily exposure to Triclosan are still conflicting.

Our recent paper demonstrated that Triclosan impairs swine luteal cell function, interfering with hormone production and cell proliferation [24]. In this context, the rationale of our present study was to increase knowledge on the potential critical effects of this molecule on granulosa cells, which represent a model of endocrine reproductive cells characterized by close functional relationships with the oocyte. In particular, the present research was performed to deepen the study of the potential effects on ovarian function. Thus, granulosa cells were isolated from swine ovarian follicles, accordingly to a validated method [25,26,27]. It is well known that the pig represents an excellent model for translational medicine due to physiological similarities with the human [28]. In the present study we employed the concentrations of Triclosan tested in our previous research [24] to study swine granulosa cell growth (BrdU incorporation and ATP production), hormone productions (17-β estradiol and progesterone secretion) and oxidative stress markers (superoxide anion, hydrogen peroxide and nitric oxide production, enzymatic and non-enzymatic scavenging activity).

## 2. Materials and Methods

All reagents, unless otherwise indicated, were acquired from Sigma Chemical Co. Ltd. (St. Louis, MO, USA), while plastic material was from Sarstedt AG&Co (Numbrecht, Germany).

### 2.1. Isolation and Culture of Granulosa Cells

Granulosa cells were isolated from swine ovaries, which had been retrieved at a slaughterhouse nearby. Ovaries were transported in a freezer bag containing cold phosphate-buffered saline solution (PBS; 4 °C) supplemented with penicillin, streptomycin and amphotericin B at concentrations of 100 lU/mL, 100 lU/mL and 2.5 µg/mL, respectively. The transport to the laboratory took place within one hour of sample retrieval.

Before processing, ovary samples were cleaned through immersion in ethanol 70% for 1 min and further sanitized through several washes in PBS [29]. All cystic or hemorrhagic follicles were discarded. Granulosa cells were collected by aspiration from follicles in their later stage of maturation (>5 mm) using a 26-gauge needle [30,31,32]. Cells were then subjected to centrifugation at 450× *g* for 10 min; ammonium chloride 0.17 M at 37 °C for 1 min was added to the cell pellet obtained to eliminate red blood cells from the preparation.

Vital staining was carried out with trypan blue dye (0.4% *w/v*) to obtain an estimated cell count. Cells were plated and cultured in a validated serum-free system employing DMEM/Ham’s F12 medium supplemented with penicillin (100 µg/mL), streptomycin (100 µg/mL), transferrin (5 µg/mL), amphotericin B (2.5 µg/mL) and sodium selenite (5 ng/mL) [33,34], indicated hereafter as CM. Granulosa cells are inhibited from luteinization by the CM, which also guarantees the maintenance of cells peculiarities.

Triclosan (catalogue number 72,779) was added to the cells in the 96-well plates right after plating at a concentration of 1, 10 or 50 µM. These concentrations had been chosen on the basis of the concentrations tested in our previous work [24] and other studies [35]. The examined concentration may be higher than a real exposure. However, bioaccumulation and biomagnification have to be taken into account when considering endocrine disruptive effects. Cells were kept for 48 h in an incubator at a constant and controlled temperature of 37 °C under humidified conditions (95% humidified air, 5% CO_2_).

### 2.2. Granulosa Cell Growth

#### 2.2.1. Cell Proliferation

Cell proliferation was evaluated by the BrdU incorporation test (Cell proliferation ELISA, BrdU, catalogue number 11647229001, Roche Diagnostics, Mannheim, Germany). After plating cells at a concentration of 10^4^/well and incubation with Triclosan in the conditions indicated above, 20 µL of BrdU was added to each well and incubated overnight. Finally, plates were centrifuged at 400× *g* for 10 min, and CM was collected. To improve antibody detection of incorporated BrdU, 200 µL of FixDenat Solution was added to each well. The presence of immune complexes was evaluated after the addition of anti-BrdU conjugated antibody. The subsequent substrate reaction was stopped and, using a Victor Reader spectrophotometer (Perkin Elmer, Groningen, The Netherland), the absorbance was quantified by measuring at a wavelength of 450 nm [36]. To quantify viable cell number, the absorbance of each sample was related to a standard curve prepared by culturing, in quintuplicate, granulosa cells at different plating densities (from 10^3^ to 10^5^ viable/200 μL) for 48 h. The curve was repeated in four different experiments. The relationship between cell number and absorbance was linear (r = 0.92). Cell number/well was estimated from the resulting linear regression equation and was used to correct experimental data. The assay detection limit was 10^3^ cell/well and the variation coefficient was less than 5% [27].

#### 2.2.2. Cell Viability Evaluation

Granulosa cell viability was estimated by means of a bioluminescent assay (ATP-lite; 1-Step 6,016,736 Perkin Elmer, Milan, Italy). The method is based on light production generated by the reaction of ATP in presence of luciferase and luciferin. The quantity of released light is directly proportional to the ATP concentration. Different numbers of viable cells, ranging from 2.5 × 10^3^ to 4 × 10^6^/100 µL, were plated to validate the method. The replicas were repeated for each curve. The test showed a concentration-dependent linear correlation between the number of cells tested and the recorded luminescence (r = 0.95).

A total of 2 × 10^5^ cells/100 µL CM were placed in 96-well plates and treated with Triclosan as detailed above. Luminescence was measured using a Victor Reader after kit reagent addition [27].

### 2.3. Granulosa Cell Steroid Production

As mentioned before, Triclosan was added to granulosa cells after seeding in 96-well plates at a concentration of 10^4^/200 µL CM supplemented with 28 ng/mL of androstenedione. Culture media were collected from each well after a two-day incubation, then they were frozen and stored at −20 °C until progesterone (P4) (DKO0036) and 17-β estradiol (E2) (DKO003) determination. To quantify P4 and E2 content, we employed a direct immunoenzymatic determination (DiaMetra s.r.l, Spello, PG, Italy) [37] based on competitive colorimetric immunological reactions. The ELISA kit for Estradiol requires a 2 h incubation of sample media at 37 °C. Subsequently, after three washes, 100 µL of TMB substrate is added. The HPR enzyme found in the bound fraction catalyzes the reaction between TMB substrate and H_2_O_2_. The sample is then left to incubate for 30 min in the dark, after which the reaction product develops a blue color. When the stop solution is added, the sample turns yellow. The hormone concentration is determined based on a 5-point calibration curve ranging from 0 to 2000 pg/mL. Absorbance values are read by the spectrophotometer at 450 nm, being the reference wavelength is 620–630 nm. The variability observed within the assay is <9%. The P4 direct immunoenzymatic determination is similar, while the concentration in the sample is calculated against a 4-point calibration curve from 0 to 40.0 ng/mL. To allow an optimal working state, the ELISA progesterone kit requires 1 h of incubation at 37 °C. Unbound antibody is then separated and 100 μL of TMB substrate is added. The sample is left in incubation for 15 min at 37 °C in the dark. The stop solution is added and the absorbance is read at 450 nm against a reference wavelength of 620–630 nm using the Victor Reader.

### 2.4. Granulosa Cell Redox Status

#### 2.4.1. Granulosa Cell Superoxide (O_2_^−^) Production

The O_2_^−^ production was evaluated by WST-1 (4-[3-(4-iodophenyl)-2-(4-nitrophenyl)-2H-5-tetrazolium]-1,3-benzene disulfonate) test (catalogue number 5015944001, Roche, Mannheim, Germany). It is built on the hydrophilic salt WST-1, which is cleaved to hydrophilic compound formazan [38]. Granulosa cells were seeded as indicated in Section 2.2 at a density of 10^4^ cells/200 µL CM. During the last 4 h of incubation, 20 µL of WST-1 was added to cells. Absorbance was recorded at 450 nm against 620 nm using Victor Reader. Coefficient of variation was always less than 3.

#### 2.4.2. Granulosa Cell Nitric Oxide (NO) Production

As indicated in Section 2.2, 2 × 10^5^ viable cells/200 μL CM were seeded with Triclosan. Subsequently, the plates were centrifuged at 400× *g* for 10 min. The superior phase was then removed and nitrite content in culture media was measured as an indicator of NO levels. This microplate method depends on a reaction with Griess reagent resulting in the production of a chromophore. Equal volumes of stock solution 1 (5% phosphoric acid) and stock solution 2 (1% sulfanilamide + 0.1% N-[naphthyl]ethylenediaminadihydrochloride) were combined to prepare the reagent freshly whenever needed. The absorbance was determined with Victor Reader using a 540 nm against 620 nm filter after the culture media had been incubated with stock solution 2. Sodium nitrite was diluted in CM to create a calibration curve that ranged from 25 to 0.39 μM [39].

#### 2.4.3. Granulosa Cell Hydrogen Peroxide (H_2_O_2_) Production

A total of 2 × 10^5^ viable cells/200 μL CM were placed in a 96-well plate and treated with Triclosan as previously described. Plates were then subjected to centrifugation at 400× *g* for 10 min and supernatants were removed. Cold Triton 0.5% + PMSF in PBS was added to each well (200 μL/well) with the aim of subjecting samples to lysis. The lysis procedure was carried out in ice bath and required 30 min of incubation. H_2_O_2_ formation was detected by an Amplex Red Hydrogen Peroxide Assay Kit (catalogue number A22188, ThermoFisher Scientific, Waltham, MA, USA); the Amplex Red reagent takes part in an oxidation reaction that involves H_2_O_2_ providing resorufin as final product. In summary, the procedure involved the preparation of a 96-well plate and the mixing of 5 μL of cell lysates with 45 μL of reaction buffer (0.05 M sodium phosphate, pH 7.4. 50 μL of Amplex Red (100 μM)/HRP (0.2 U/mL) reagent working solution, which was then added to each well. The plate was subsequently incubated at room temperature for 30 min, away from direct light. At the end of the incubation period, the plate was read and the results interpreted with reference to a standard H_2_O_2_ curve ranging from 0.39 to 50 μM. The absorbance was determined with Victor Reader using a 540 nm filter [40].

#### 2.4.4. Non-Enzymatic Scavenging Activity

Antioxidant molecules in an organic sample can reduce ferric-tripiridyltriazine (Fe^3+^ TPTZ) to a ferrous form (Fe^2+^ TPTZ). This potentiality can be evaluated with Ferric Reducing Activity of Plasma assay (FRAP), a colorimetric test that measures Fe^2+^ spectrophotometrically based on the development of the colored complex with 2,4,6-Tris(2-pyridyl)-s-triazine (Fe^2+^ TPTZ). The TPTZ reagent preparation was performed daily. The preparation involves mixing 25 mL of acetate buffer, 2.5 m L of 2,4,6-Tris(2-pyridyl)-s-triazine (TPTZ) 10 mM in HCl 40 mM and FeCl_3_−6H_2_O solution.

The 2 × 10^5^ cells/200 μL CM were placed in 96-well plates and treated with Triclosan. Plates were then subjected to centrifugation at 400× *g* for 10 min and supernatants were removed. Cold Triton 0.5% + 200 μL PMSF in PBS was added to each well to perform cell lysis procedure, which was carried out in ice bath for 30 min. Fe ^3+^ TPTZ was added to 40 μL of cell lysates and the solution was incubated for 30 min at a temperature of 37 °C. The absorbance of Fe^2+^ TPTZ was determined afterwards using the Victor Reader at 595 nm. A standard curve of absorbance against FeSO_4_−7H_2_O standard solution was plotted after calculating the ferric reducing ability of cell lysates.

#### 2.4.5. Enzymatic Scavenging Activity

The 2 × 10^5^ viable cells/200 μL CM were placed in 96-well plates and treated with Triclosan as previously described. Plates were then subjected to centrifugation at 400× *g* for 10 min and supernatants were removed. Cold Triton 0.5% + 200 μL PMSF in PBS was added to each well to perform the cell lysis procedure, which was carried out in ice bath for 30 min. Cell lysates were used to assay determine superoxide dismutase (SOD), glutathione peroxidase (GSH) and catalase (CAT) activities.

The SOD activity was detected by a SOD Assay Kit (catalogue number 19160, Sigma Chemical Co. Ltd., St. Louis, MO, USA). The kit was performed on 20 µL of cell lysate not subjected to dilution and results were reported to a standard SOD curve ranging from 0.156 to 20 U/mL. The colorimetric assay allows the measurement of the amount of formazan developing from the reaction between a tetrazolium salt and a superoxide anion (O_2_^−^), produced by the reaction of an exogenous xanthine oxidase. The remaining O_2_^−^ is an indirect hint of the endogenous SOD activity. The absorbance was determined with Victor Reader reading at 450 nm against 620 nm [41].

The GSH activity was measured by an Amplex Red Peroxidase Assay Kit (catalogue number A22188, ThermoFisher Scientific, Waltham, MA, USA), which is based on the identification of an oxidation product of the reaction between H_2_O_2_ given in excess and the Amplex Red reagent: resorufin. A total of 5 µL of cell lysates was dispensed in each well of a 96-well plate and mixed with 45 µL of reaction buffer (0.05 M sodium phosphate, pH 7.4). A total of 50 µL of Amplex Red reagent (100 µM)/H_2_O_2_ (2 mM) working solution was then added to each well. Thereafter, the plates were incubated at room temperature for 30 min, away from direct light. The reading was performed against a standard curve of GSH ranging from 0.078 to 10 mU/mL. The absorbance was determined with a Victor Reader using a 540 nm filter [40].

An Amplex Red Catalase Assay Kit (A22180 ThermoFisher Scientific, Waltham, MA, USA) was used to detect CAT activity. The kit is based on the identification of an oxidation product of the reaction between H_2_O_2_ given in excess and the Amplex Red reagent in presence of horseradish peroxidase: resorufin. The kit was performed on 25 µL of cell lysate subjected to a 1:10 dilution. Cell lysates were seeded in each well and mixed with 25 µL of H_2_O_2_ (40 µM). Plates were then incubated for 30 min at room temperature. A total of 50 µL of Amplex Red reagent (100 µM)/HRP (0.4 U/mL) working solution was then added to each well. The plates were subsequently incubated at room temperature for 30 min, away from direct light. The reading was performed against a standard curve of CAT ranging from 62.5 to 1000 mU/mL. The absorbance was determined with a Victor Reader using a 540 nm filter [40].

### 2.5. Statistical Analysis

Five replicates of each experiment were carried out. Each time, the ovaries were collected from 40 gilts. Six replicates of each treatment with Triclosan at different concentrations were performed. Results are expressed as mean ± SEM. Statgraphics software 5 Plus (STC Inc., Rockville, MD, USA) was used to carry out the ANOVA test. Scheffè’ F test was employed for multiple comparisons whenever a significant difference (*p* < 0.05) was found.

## 3. Results

### 3.1. Effect of Triclosan on Swine Granulosa Cells Growth

**Figure 1:**
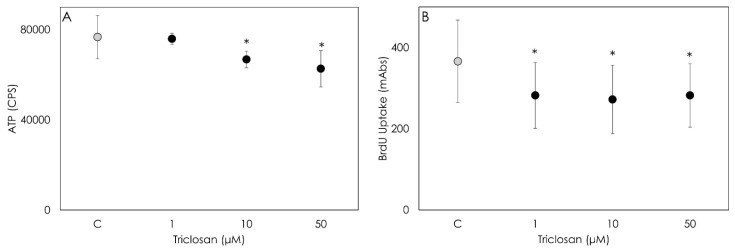
Result of ATP (**A**) and 5-bromo-2′deoxyuridine (BrdU) (**B**) tests carried out on porcine granulosa cells following treatment with Triclosan (1, 10 or 50 µM) for 48 h. The tests evaluate the metabolic activity and proliferation of treated cells, respectively. Data are expressed as counts per second (CPS) (**A**) and on milli-Abs units (**B**) and represent the mean ± SD of six replicates/treatment repeated in five experiments (n = 30). Asterisks on points indicate that data show significant differences (*p* < 0.001).

The ATP production evaluation revealed that Triclosan impairs cell metabolic activity at high dosages. In fact, a significant decrease (*p* < 0.001) was observed after the 48 h treatment with 10 and 50 µM of Triclosan, while a 1 µM concentration was ineffective (Figure 1A). The cells exposed for 48 h to a 1, 10 or 50 µM Triclosan treatment displayed a significant decrease in cell proliferation evaluated by BrdU incorporation (Figure 1B). No dose-dependent response was observed, since no differences were detected among the concentrations tested.

### 3.2. Effect of Triclosan on Porcine Granulosa Cell Steroidogenesis

**Figure 2:**
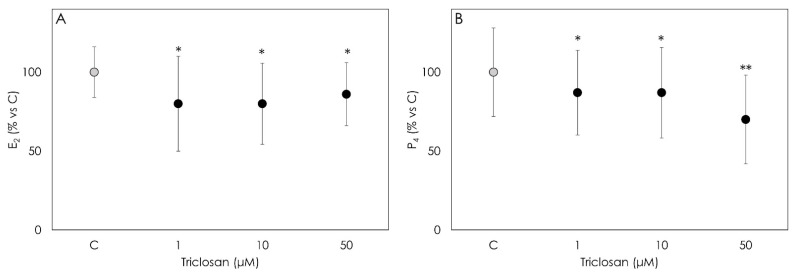
Results of the production of 17β estradiol (E2) (**A**) and progesterone (**B**) by porcine granulosa cells treated for 48 h with Triclosan (1, 10 or 50 µM) detected by ELISA assay. Data are expressed as % vs. control and represent the mean ± SD of six replicates/treatment repeated in five different experiments (n = 30). Asterisks on points indicate that data show significant differences (*p* < 0.001).

Data obtained document that Triclosan impairs granulosa cell steroidogenesis. In particular, both progesterone and 17-β estradiol were significantly inhibited (*p* < 0.001) by the treatment (Figure 2A,B). As for P4 secretion, the strongest inhibitory effect was observed in cells treated with 50 µM Triclosan (*p* < 0.001).

### 3.3. Effect of Triclosan on Porcine Granulosa Cell Redox Status

**Figure 3:**
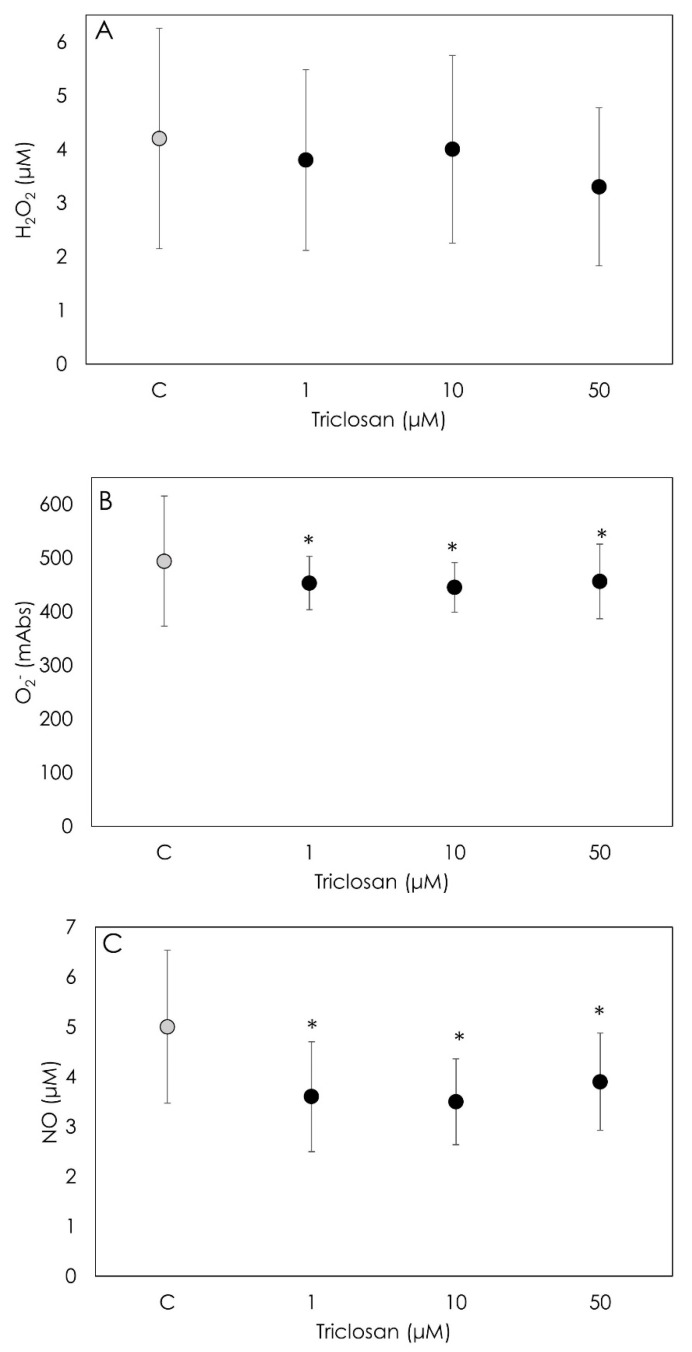
Results of hydrogen peroxide (H_2_O_2_) (**A**), superoxide anion (O_2_^−^) (**B**) and nitric oxide (NO) (**C**) generation using colorimetric assay on porcine granulosa cells treated for 48 h with Triclosan (1, 10 or 50 µM). Data are expressed as milliAbs units (**B**) and as µM (**A**,**C**) and represent the mean ± SD of six replicates/treatment repeated in five different experiments (n = 30). Asterisks on points indicate that data show significant differences (*p* < 0.05).

We did not observe a significant effect of Triclosan treatment on the H_2_O_2_ production by granulosa cells at any concentration tested, as shown in Figure 3A. On the contrary, both O_2_^−^ and NO levels were strongly inhibited (*p* < 0.001) by Triclosan treatment at all the tested concentrations. No significant differences were observed among dosages (Figure 3B,C).

**Figure 4:**
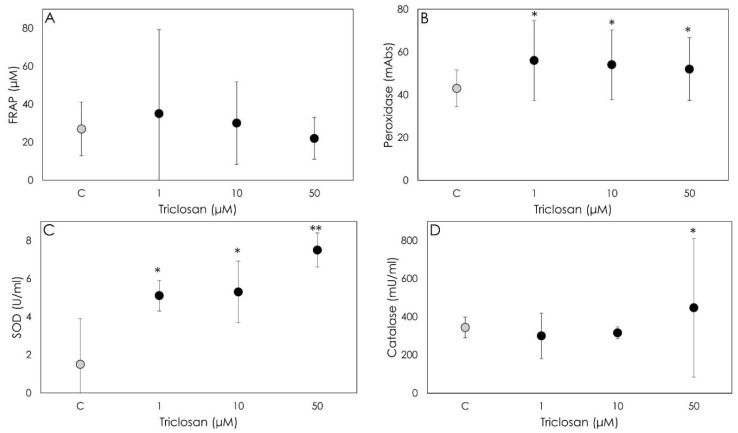
Results of non-enzymatic scavenging activity by porcine granulosa cells treated for 48 h with Triclosan (1, 10 or 50 µM) using the FRAP assay ((**A**); µM), on peroxidase activity ((**B**); milliAbs), superoxide dismutase activity ((**C**); U/mL) and on catalase activity ((**D**); mU/mL). Data represent the mean ± SD of six replicates/treatment repeated in five different experiments (n = 30). Asterisks on points indicate that the data show significant differences (*p* < 0.05).

Triclosan did not modify the non-enzymatic antioxidant power and therefore scavenging activity (Figure 4A). On the contrary, all the enzymatic scavengers were significantly (*p* < 0.05) stimulated; in particular, Triclosan showed a relevant stimulatory effect on GSH and SOD at all the examined concentrations (*p* < 0.05) (Figure 4B,C). As for CAT, increased levels were observed only after the Triclosan treatment at the highest concentration (Figure 4D).

## 4. Discussion

While Triclosan’s critical role has been demonstrated both in reproductive processes [23,42,43] and fertility [21,44], its effect on ovarian function have been scarcely investigated. In a previous study, we demonstrated that this substance disrupts the functionality of cultured cells isolated from the swine corpus luteum, the transient endocrine organ that develops after ovulation [24]. The present research was undertaken to deepen the knowledge of its potential effect on the ovarian district focusing on granulosa cells in the ovarian follicle, which represent the main endocrine cells in the follicular unit. To our knowledge, the influence of Triclosan on granulosa cells has only been investigated in a human granulosa-like tumor cell line, KGN [45], and in primary rat granulosa cell culture [35]. Therefore, in order to collect information on primary granulosa cells we isolated and cells from the pig, which represents a reliable animal model due to its well-known value in translational medicine [28]. We studied the effects produced by Triclosan on the main functional activity of granulosa cells, i.e., growth, steroidogenesis and redox status markers [27], after selecting the concentrations on the bases of previous reports [14,24,35]. The Triclosan tested concentrations were chosen on the basis of the assays in human matrices. Urine is generally accepted as the matrix of choice for the biomonitoring of Triclosan exposure, while other biological fluids, such as blood, breast milk and amniotic fluid, are rarely used [46,47,48]. The cumulative exposure to Triclosan could be evaluated by the analysis of human nails [49].

The presented data confirm in our previous observations in luteal cells [24] as regards an inhibitory action of Triclosan on cell metabolic activity and proliferation. As previously demonstrated in rat placental cells [50], the negative effect could be due to an involvement of Akt-mTOR-p70S6K signaling; research is ongoing to assess this hypothesis. Also, rat granulosa cell viability [35] as well as viability and ATP production in the human granulosa-like tumor cell line KGN [45] were inhibited by Triclosan. It is well known that granulosa cell proliferation is essential during follicle growth and development [51] until ovulation [52]. During growth, the follicle epithelium expands by increasing the number of GC layers forming the follicular epithelium surrounding the oocyte and then they expand laterally. Therefore, since granulosa cell growth and proliferation can be considered as an important marker of ovarian follicle development, the inhibition induced by Triclosan represents a very critical effect impairing the physiological follicle activity.

Endocrine-disrupting chemicals are defined as exogenous substances that can affect the physiological endocrine functions; in fact, by mimicking or antagonizing endogenous hormones, or disrupting the normal synthesis and metabolism of endogenous hormones and their related receptor functions, they can affect the body’s function [53]. In particular, these substances impair hormonal homeostasis and result in estrogen signaling changes. It is well known that granulosa cells preserve and rear oocytes, secrete steroid hormones such as estrogen and progesterone, and thus furnish a suitable microenvironment for follicular development. The correct balance of ovarian steroid hormones is pivotal for the normal development and maturation of follicles. Although Triclosan has been generally recognized as an endocrine disruptor chemical [54,55], at present its effects on ovarian cell steroidogenesis appear more difficult to define. In our previous study on luteal cells [24], we observed a biphasic effect caused by Triclosan: at the lower concentrations it caused a stimulation, while it produced an inhibition at the higher concentration tested. This finding is in accordance with the results previously documented [56]. A stimulatory effect on steroidogenesis has also been reported by Du et al. [45] in the granulosa-like tumor cell line KGN, and by Chen et al. [35] in primary rat granulosa cells. It should be noted that both studies were performed by culturing granulosa cells with serum, a method that results in cell luteinization [57]. In general, granulosa cells suffer from an inadequate culture method and their luteinization represents a real problem, mainly resulting from the presence of serum in the culture medium. To avoid this issue, we developed a serum-free system [58] built on similar homologue [59] or heterologue [60,61] serum-free species. The adequacy of our cell culture system, which guarantees the maintenance of cultured cell function, is supported by our previous work. In fact, we documented that porcine-cultured granulosa cells maintained in serum-free medium for 48 h preserve their ability to replicate after both FSH and IGF-I stimulation. Our results also show the maintenance of basal estradiol production by granulosa cells grown in this culture system. Indeed, the cultured cells displayed an estrogenic dose-dependent response to physiological FSH doses. In these culture conditions, granulosa cells can grow in a more physiological way since they don’t adhere to plastic in a fibroblast-like fashion and retain their cuboidal morphological appearance. Therefore, the swine granulosa cell culture in the present research has been set up by employing a well-validated serum-free culture method that guarantees the maintenance of granulosa cell features [61]. In our culture conditions, Triclosan displayed a disruptive effect on granulosa cell steroidogenesis by inhibiting the production of both P4 and E2. This effect should be taken into account, since changes in vital processes that support hormonal ovarian function can lead to ovarian pathologies and could be responsible for anovulation and infertility.

Increasing evidence exists that endocrine disruptors are responsible for oxidative stress [62,63]. Since it has been clearly demonstrated that redox balance is essential for an adequate ovarian follicle function resulting in a successful ovulation [64], we sought to consider the potential effect of Triclosan on the main parameters involved in this crucial aspect. In a previous study [24], we demonstrated that Triclosan can affect the luteal cell redox status. Our present results show an imbalance between cellular oxidant species production (ROS) and antioxidant cell capability; in fact, O_2_^−^ and NO, crucial molecules in granulosa cell signaling [65], were reduced while antioxidant scavenger enzyme activities were potentiated. In our previous study performed in luteal cells [24], we showed that Triclosan inhibits enzymatic scavenger activity while inducing ROS increase, thus indicating a weaker defensive power in terminally differentiated cells. In fact, luteal cells are terminally differentiated cells, contrarily to granulosa cells, which possess instead a differentiative power [51]. It should be noted that ROS can either be harmful or beneficial, depending on their levels. In optimal circumstances, the amount of ROS produced and scavenging rates are similar, thanks to the defensive mechanisms of enzymatic and non-enzymatic antioxidants. Superoxide dismutase, catalase and glutathione peroxidase are among the most common enzymatic defense strategies present in living organisms and are directly involved in the removal of ROS. The modulation of antioxidant mechanisms is based not only on the oxidative state of the cell, but also on other factors such as hormones. To our knowledge, the effect of Triclosan on granulosa cell oxidative stress markers has never been documented, but this disruptive effect deserves further research. In addition, the inhibition of NO generation represents a severe risk factor for the maintenance of a physiological follicular function, since NO plays a pivotal role in driving local angiogenesis, a biological event fundamental for ovulation [65]. At present, the negative impact of the suppression of NO levels by Triclosan appears still speculative and further studies are in course to unravel the potential links that could clarify this issue with a more detailed and comprehensive view.

Since most of the effects resulted from treatment with the lowest concentration tested, it appears interesting to verify cell responses to Triclosan doses lower than 1 µM. Actually, this evaluation could clarify crucial aspects involved with real exposure to this disrupting agent. Furthermore, it is essential to investigate the Triclosan mechanism of action, performing studies to explore the impact at gene level.

## 5. Conclusions

The data obtained show that Triclosan hinders the main functions of granulosa cells, by reducing hormone production, which is essential for the maturation process of the oocyte within the ovarian follicle, by inhibiting granulosa cell growth, which is crucial for follicle development, and by impairing redox balance, which is a crucial hallmark until ovulation. These results, therefore, appear to support the potential critical effects of Triclosan on reproductive function and suggest recommending the discontinued use of Triclosan in sanitizers and personal care products. Since most of the effects seem to be exerted by the lowest dose tested, mostly without a clear dose–effect response, further research is required to explore Triclosan concentrations lower than 1 µM. In addition, it appears of the outmost importance to focus future studies with careful attention to the effects produced at the gene level.

## Data Availability

Data will be available upon request.
